# High-throughput sequencing technology to reveal the composition and function of cecal microbiota in Dagu chicken

**DOI:** 10.1186/s12866-016-0877-2

**Published:** 2016-11-04

**Authors:** Yunhe Xu, Huixin Yang, Lili Zhang, Yuhong Su, Donghui Shi, Haidi Xiao, Yumin Tian

**Affiliations:** 1 Department of Animal Husbandry & Veterinary Medicine, Liaoning Medical University, Jinzhou, Liaoning 121000 China; 2 Department of Veterinary Medicine, Nanjing Agricultural University, Nanjing, Jiangsu 210095 China; 3 Department of Food Science, Liaoning Medical University, Jinzhou, Liaoning China

**Keywords:** High-throughput sequencing technology, Feeding modes, Cecal microbiota, Composition and function

## Abstract

**Background:**

The chicken gut microbiota is an important and complicated ecosystem for the host. They play an important role in converting food into nutrient and energy. The coding capacity of microbiome vastly surpasses that of the host’s genome, encoding biochemical pathways that the host has not developed. An optimal gut microbiota can increase agricultural productivity. This study aims to explore the composition and function of cecal microbiota in Dagu chicken under two feeding modes, free-range (outdoor, OD) and cage (indoor, ID) raising.

**Results:**

Cecal samples were collected from 24 chickens across 4 groups (12-w OD, 12-w ID, 18-w OD, and 18-w ID). We performed high-throughput sequencing of the 16S rRNA genes V4 hypervariable regions to characterize the cecal microbiota of Dagu chicken and compare the difference of cecal microbiota between free-range and cage raising chickens. It was found that 34 special operational taxonomic units (OTUs) in OD groups and 4 special OTUs in ID groups. 24 phyla were shared by the 24 samples. Bacteroidetes was the most abundant phylum with the largest proportion, followed by Firmicutes and Proteobacteria. The OD groups showed a higher proportion of Bacteroidetes (>50 %) in cecum, but a lower Firmicutes/Bacteroidetes ratio in both 12-w old (0.42, 0.62) and 18-w old groups (0.37, 0.49) compared with the ID groups. Cecal microbiota in the OD groups have higher abundance of functions involved in amino acids and glycan metabolic pathway.

**Conclusion:**

The composition and function of cecal microbiota in Dagu chicken under two feeding modes, free-range and cage raising are different. The cage raising mode showed a lower proportion of Bacteroidetes in cecum, but a higher Firmicutes/Bacteroidetes ratio compared with free-range mode. Cecal microbiota in free-range mode have higher abundance of functions involved in amino acids and glycan metabolic pathway.

**Electronic supplementary material:**

The online version of this article (doi:10.1186/s12866-016-0877-2) contains supplementary material, which is available to authorized users.

## Background

Chickens have proportionally smaller intestines and shorter transit digestion times than mammals, but do not appear to any less efficient at digestion than their mammalian counterparts [1, 2]. Their digestive system is adapted to extract energy from difficult to digest food sources. This may be explained, in part, by the fact that the chicken gastrointestinal tract is home to a complex microbial community, the chicken gut microbiota, which underpins the links between diet and health [3, 4]. The host is unable to digest and utilize the complicated polysaccharide substance from the feedstuff in the absence of microbial fermentation [5]. Particularly relevant to the intensive farming of chickens is the cecum’s role in digestion of non-starch polysaccaharides NSPs [6], which are found in the grains used in commercial chicken feed. The gut microbiota has one of the highest cell densities for any ecosystem and ranges from 10^7^ to 10^11^ bacteria per g of gut content in poultry [7]. The most densely populated microbial community within the chicken gut is found in the ceca, a pair of blind-ended sacs that open off the large intestine [8]. This microbiota is also home to a rich collection of genes, the chicken gut microbiome, likely to include many sequences of scientific interest and biotechnological potential [4]. The coding capacity of microbiome vastly surpasses that of the host’s genome, encoding biochemical pathways that the host has not developed.

An optimal gut microbiota can increase agricultural productivity, as evidenced by the ability of antibiotics to promote growth in chickens [9]. Studies on rumen microbe in ruminants have revealed that *Ruminococcus* and *Fibrobacter* species are important members of the rumen microbial community that enable the host to degrade and utilize fibrous plant materials efficiently as nutrients [10–12]. As a result, animal productivity has been improved through refining the animals’ ability to degrade fiber by these microorganisms. Energy and nutrient extraction from feed requires interplay between the biochemical functions provided by the chicken and the microbiota present within the gastrointestinal tract (GIT). Highly productive chickens have been developed by selection for elite genetic traits; it is possible that in the future, gains in productivity and health outcomes could be influenced by selection of elite GIT microbiota [13]. Therefore, studies on the composition and functions of gut microbiota in animals raised in different feeding modes is significant for the improvement of feedstuff efficiency and animal productivity. At present, our ability to culture intestinal bacteria is limited, and hence, there is a need to profile and investigate this community using culture-independent techniques. Culture-independent analysis of the chicken cecal microbiota estimated 900 species of bacteria in 100 genera existing in the cecum of chickens, with most of them belonging to uncategorized genera [7, 14]. Previous studies have shown that the caeca microbial communities were more diverse in comparison to ilea [15]. Left and right ceca of chickens are harbouring similar bacterial communities [2]. But, the composition and function of cecal microbiota under different feeding mode are unknown.

Consumer interest in free-range and organic poultry is growing. The meat of the outdoor chickens had more protein than the indoor chickens [16]. Dagu chicken is a well-known local breed in China. Dagu chicken is native to Zhuanghe City, Liaoning and is free-range. This chicken has been called Cao Chicken (*cao* means grass in Chinese) because of the favorable living environment and fine feed resources of water and grass. Whether living habits influence the formation of gut microflora in Dagu chicken are unknown.

This study aims to explore the composition and function of cecal microbiota in Dagu chicken under two feeding modes, free-range and cage raising. Thus, providing base informations for designing high efficiency feed formula, developing applicable probiotics and regulating chicken meat quality.

## Methods

### Chicken farm and sampling

Zhuanghe City is located in the south end of the Liaodong peninsula. Its location, with coordinates N39.32′–40.5′, E122′–124.5′, indicates a typical mountainous hilly terrain. Dagu chicken in free-range farming is a traditional feeding in Zhuanghe and relies on abundant rivers and flourish pasture. Thus, this study was carried out in Zhuanghe Dagu chicken breeding center.

A total of 1000 1-day-old male Dagu chickens were selected. The chickens were raised in plastic mesh floors (80 cm above ground) for 6 weeks. The chickens were provided access to feed and water ad libitum. The house temperature was maintained at 35 °C during the first week, and it was reduced 2 °C per week until reaching the temperature of 23 °C. Six weeks later, 300 chickens with similar weights were randomly selected. Among them, 150 chickens were raised outside, which are in the outdoor group (OD group), while the other 150 chickens were raised inside their respective cages (50 cm × 50 cm × 50 cm,80 cm above ground) which are in the indoor group (ID group). The house temperature was maintained at 23 °C. The chickens were provided access to feed and water ad libitum. The difference of body weight between the two groups was not significant (*P* >0.05). Two groups were given the same compound feed (Additional file 1) as well as other environmental factors. The difference is for the OD group, each chicken in the OD group was let out every 5 am for self-help feeding in >30 m^2^ area, where abundant water and grasses are found. The chickens were given supplementary feed at 1 pm, and kept indoors at 7 pm. When the chickens were 12 weeks old and 18 weeks old, weighed one by one, six of them with an average weight were randomly selected in each group,and then slaughtered. The cecum contents removed, preserved in liquid nitrogen, used for DNA extraction and PCR amplification. These samples were divided into four groups, namely, 12-w OD group, 12-w ID group, 18-w OD group, and 18-w ID group.

### Gut microbes 16S rRNA sequencing

Microbial genomic DNA was extracted from cecal content samples by using the TIANGEN DNA stool mini kit (TIANGEN, cat#DP328) according to the producer’s instructions (http://www.tiangen.com/asset/imsupload/up0921879001368428871.pdf). Variable region of 16S rRNA V4 was amplified using its universal primer sequence 520 F: AYTGGGYDTAAAGNG; 802R: TACNVGGGTATCTAATCC [17]. The PCR conditions were as follows: initial denaturation at 98 °C for 5 min; 98 °C denaturation for 30 s, 50 °C annealing for 30 s, and 72 °C extension for 30 s, which is repeated for 28 cycles; and a final extension at 72 °C for 5 min. PCR production was purified using QIAGEN Quick Gel Extraction Kit (QIAGEN, cat# 28706). PCR production from each sample was applied to construct a sequencing library by using Illumina TruSeq DNA Sample Preparation Kit (library was constructed using TruSeq Library Construction Kit). For each sample, barcoded V4 PCR amplicons were sequenced by the Illumina MiSeq PE250 platform.

Sequence reads were removed if sequence length was shorter than 150 bp, if average phred score was lower than 20, if containing ambiguous bases, if homopolymer run exceeded 6, or if there were mismatches in primers. Afterward, the sequences passed the quality filter that were assembled by Flash (http://www.genomics.jhu.edu), which required that the overlap of read 1 and read 2 ≥ 10 bp, and without any mismatches. The reads which could not be assembled were discarded. Chimera sequences were removed using UCHIME in mothur (version 1.31.2, http://www.mothur.org/). Amplification and sequencing of 16S rRNA v4 variable region was completed by Personal Biotechnology Co., Ltd. (Shanghai, China).

### OTU clustering and statistical analysis

Sequences clustering was performed by uclust algorithm in QIIME (http://qiime.org/scripts/pick_otus.html), and clustered into operational taxonomic units (OTUs). The longest sequence in each cluster was selected as the representative. Taxonomy of each OTU was assigned by blasting the representative sequence against Greengenes reference database (Release 13.8, http://greengenes.secondgenome.com/). Unknown archaeal or eukaryotic sequences were filtered and removed. Ace, Chao, Simpson index were calculated using summary.single command in MOTHUR. A Venn diagram of between-group OTU was generated through R. The relative abundance of OTUs or taxa was compared between samples.

Diversity index data were analyzed statistically using analysis of variance (ANOVA) and significant differences between group means were determined using the least significant difference (LSD) test. Data of body weight and abundance at the phylum level between groups were analyzed statistically using *T* test. All values for diversity index and body weight are expressed as means ± standard errors (SE). Non-metric multidimensional scaling (NMDS) plots of sequence read abundance were generated with Vegan in R. All statistical analyses were performed using the SPSS 16.0 software.

### Microbial function prediction

Functional genes were predicted through PICRUSt according to the abundance of OTU level [18]. The OTUs were mapped in gg13.5 database at 97 % similarity by QIIME’s command “pick_closed_otus”. The abundance of the OTUs was normalized automatically by using 16S rRNA gene copy numbers from known bacterial genomes in the Integrated Microbial Genomes (IMG). The predicted genes and their function were aligned to the Kyoto Encyclopedia of Genes and Genomes (KEGG) database and the differences among groups were compared using STAMP (http://kiwi.cs.dal.ca/Software/STAMP) [19]. Two-side Welch’s *t*-test and Benjamini–Hochberg FDR correction were employed in the two-group analysis. The relative abundance of KEGG metabolic pathways is referred to as a metabolic profile.

## Results

### OTU clustering and annotation

**Fig. 1:**
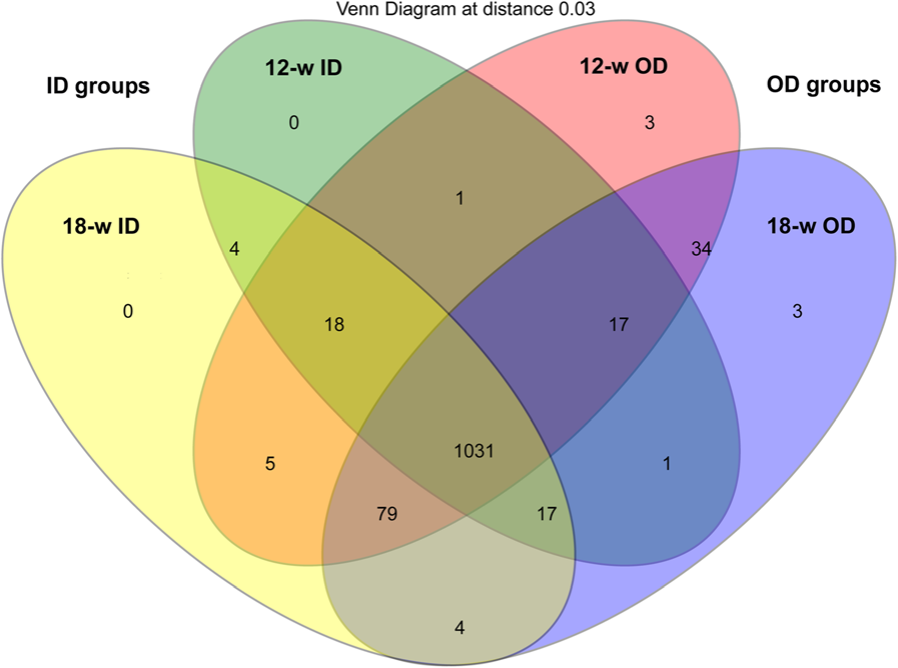
Shared OUT analysis of the different groups. Numbers below groups indicate the number of OTUs within each sector. The number of species in 12-w OD is 1188; The number of species in 12-w ID is 1089; The number of species in 18-w OD is 1186; The number of species in 18-w ID is 1158; The number of species shared between 12-w OD and 12-w ID is 1067; The number of species shared between 12-w OD and 18-w OD is 1161; The number of species shared between 12-w OD and 18-w ID is 1133; The number of species shared between 12-w ID and 18-w OD is 1066; The number of species shared between 12-w ID and 18-w ID is 1070; The number of species shared between 18-w OD and 18-w ID is 1131; The number of species shared between 12-w OD, 12-w ID and 18-w OD is 1048; The number of species shared between 12-w OD, 12-w ID and 18-w ID is 1049; The number of species shared between 12-w OD, 18-w OD and 18-w ID is 1110; The number of species shared between 12-w ID, 18-w OD and 18-w ID is 1048; The total richness of all the groups is 1217

The trimmed and assembled sequences were clustered at 97 % similarity by calling uclust from Qiime. 1217 OTUs were obtained through database alignment by blast in Qiime. The total of OTUs obtained in each group were as follows: 1188 in the 12-w OD group, 1089 in the 12-w ID group, 1186 in the 18-w OD group, and 1158 in the 18-w ID group (Fig. 1). Figure 1 shows 34 special OTUs in OD groups (including 12-w OD and 18-w OD) and 4 special OTUs in ID groups (including 12-w OD and 18-w OD). The number of OTUs in each group slightly changed in the OD groups, whereas that increased in the ID groups within days. The diversity of cecal microbiota in OD groups can be established earlier. The Chao and ACE in the 12-w OD group were significantly higher (*P* < 0.05) than those in the three other groups, but the Simpson in the OD groups was significantly lower (*P* < 0.05) than that in the ID groups. These results revealed that the richness of cecum microorganism in the 12-w OD group was higher than those in the three other groups, the evenness of cecum microorganism in the ID groups was higher than those in the OD groups (Table 1).

**Table 1:** Diversity index

Group (*n* = 6)	12-w		18-w	
	OD	ID	OD	ID
Chao	4128 ± 733^b^	2273 ± 145^a^	2798 ± 223^a^	2814 ± 209^a^
ACE	5877 ± 1180^b^	3101 ± 218^a^	3884 ± 354^a^	3862 ± 323^a^
Simpson	0.037 ± 0.0087^ab^	0.088 ± 0.0231^c^	0.030 ± 0.0024^a^	0.064 ± 0.0141^bc^

Means with the same superscript within the same row are not significantly different,with the different small letters are significant; the means difference is significant at the 0.05 level

### Differences of body weight and cecal microbiota in chickens raised in different feeding modes

In this study, chicken body weight in different feeding modes has obvious differences. Chicken body weight in the ID group was significantly higher than that in the OD group both 12-w or 18-w stage (Table 2).

**Table 2:** Body weight

Group	Body weight g	
	OD	ID
12-W (*n* = 150)	1932.40 ± 13.24 ^a^	2065.97 ± 11.36 ^b^
18-W (*n* = 144)	2584.44 ± 18.39 ^a^	2804.24 ± 15.76 ^b^

Means with the different small letters within the same row are significantly; The means difference is significant at the 0.05 level

**Fig. 2:**
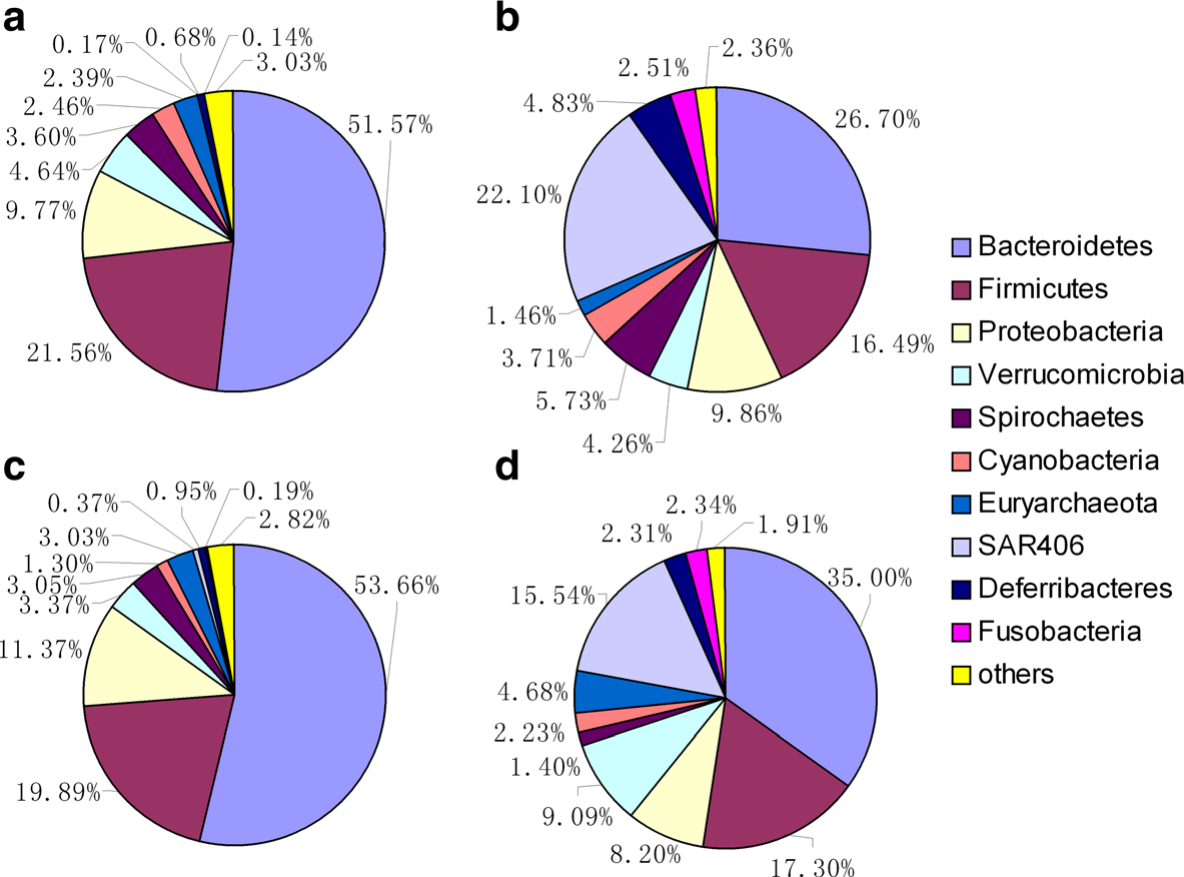
Distribution of the cecum microbiota composition at the rank of phylum. **a** 12-w OD group. **b** 12-w ID group. **c** 18-w OD group. d, ID group. The proportions of each phylum in the 12-w OD and 12-w ID groups are as follows: Bacteroidetes: 51.57 %, 26.7 %; Firmicutes: 21.56 %, 16.49 %; Proteobacteria: 9.77 %, 9.86 %; Verrucomicrobia: 4.64 %, 4.26 %; Spirochaetes: 3.60 %, 5.73 %; Cyanobacteria: 2.46 %, 3.71 %; Euryarchaeota: 2.39 %, 1.46 %; SAR406: 0.17 %, 22.10 %; Deferribacteres: 0.68 %, 4.83 %; and Fusobacteria: 0.14 %, 2.51 %. The proportions of each phylum in the 18-w OD group and 18-w ID group are as follows: Bacteroidetes: 53.66 %, 35.00 %; Firmicutes:19.89 %, 17.30 %; Proteobacteria: 11.37 %, 8.20 %; Verrucomicrobia: 3.37 %, 9.09 %; Spirochaetes: 3.05 %, 1.40 %; Cyanobacteria: 1.30 %, 2.23 %; Euryarchaeota: 3.03 %, 4.68 %; SAR406: 0.37 %, 15.54 %; Deferribacteres: 0.95 %, 2.31 %; and Fusobacteria: 0.19 %, 2.34 %

A total of 24 phyla were shared by the 24 samples. Bacteroidetes was the most abundant phylum with the largest proportion, followed by Firmicutes and Proteobacteria (Fig. 2). Three significant differences (*P* < 0.05) in the 12-w groups and five significant (*P* < 0.05) differences in the 18-w groups were found (Table 3).

**Table 3:** Comparisons for abundance at the phylum level

Phylum	12-w relative fold change (log_2_^OD/ID^)	*P* value	18-w relative fold change (log_2_^OD/ID^)	*P* value
Actinobacteria	1.3201	0.029	1.4615	0.069
Bacteroidetes	0.9498	0.000	0.6163	0.007
Elusimicrobia	1.0627	0.089	2.6878	0.033
Fusobacteria	−4.1746	0.054	−3.6384	0.043
SAR406	−7.0588	0.011	−5.4099	0.022
Tenericutes	0.4183	0.436	1.0385	0.045

Spirochaetes had dynamic changes in the ID groups; its proportion was 5.73 % in the 12-w ID group, but it reduced to 1.4 % in the 18-w ID group. However, the proportion had a slight change in the OD groups (3.6 %, 3.05 %).

In the 12-w groups, Bacteroidetes, Firmicutes and Proteobacteria accounted for 83 % and 53 %, and Bacteroidetes for 52 % and 26.7 % in the OD and ID groups, respectively. In the 18-w groups, the three phyla accounted for 84.9 % and Bacteroidetes accounted for 53.66 % in the OD group, which exhibited a slight difference from that in the 12-w OD group. The proportion was 60.5 %, and Bacteroidetes had the largest share of 35 % in the ID group.

SAR406 mainly existed in the ID groups, accounting for 22.1 % in the 12-w group and 15.54 % in the 18-w group. SAR406 accounted for 0.17 % in the 12-w group and 0.37 % in the 18-w group in the OD groups, respectively.

At the genus level we detected 60 genera. 10 genera were significantly different (*P* < 0.01) between the 12-w OD and 12-w ID groups (Additional file 2), 6 genera were significantly different (*P* < 0.01) between the 18-w OD and 18-w ID groups (Additional file 3).

**Fig. 3:**
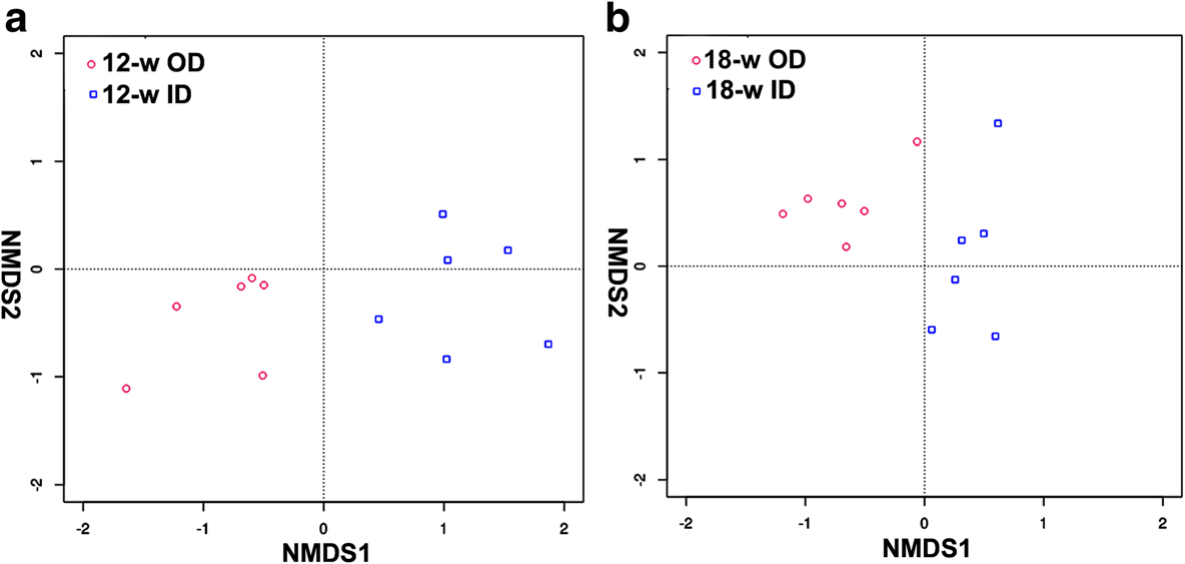
NMDS ordination. **a** 12-w OD group and 12-w ID group. **b** 18-w OD group and 18-w ID group. NMDS plots demonstrate that free-range and cage ceca are harbouring different bacterial communities

NMDS results showed the difference in microorganism distributions in the four groups. The distribution was evidently different in the OD groups compared with that in the ID groups (Fig. 3). The microorganisms in the OD groups concentrated on one group whereas those in the ID groups concentrated on another. Numerical values in correlation analysis revealed that the cecal microbiota in the 12-w OD groups were quite different from those in the 12-w ID group (0.5729). However, the cecal microbiota in the 18-w OD group were remarkably similar to those in the 18-w ID group (0.9626) (Table 4). The results show that the richness and evenness of cecal microbiota in chickens raised in cages were noticeably different from those in chickens from free-range farming, especially at 12 weeks.

**Table 4:** Correlation between groups for genus abundance

	12-w ID(*N* = 6)	18-w OD (*N* = 6)	18-w ID (*N* = 6)
12-w OD(*N* = 6)	0.5729	0.9936	0.9867
12-w ID (*N* = 6)		0.5767	0.6792
18-w OD (*N* = 6)			0.9626

Six samples from each group were used to calculate correlation

**Fig. 4:**
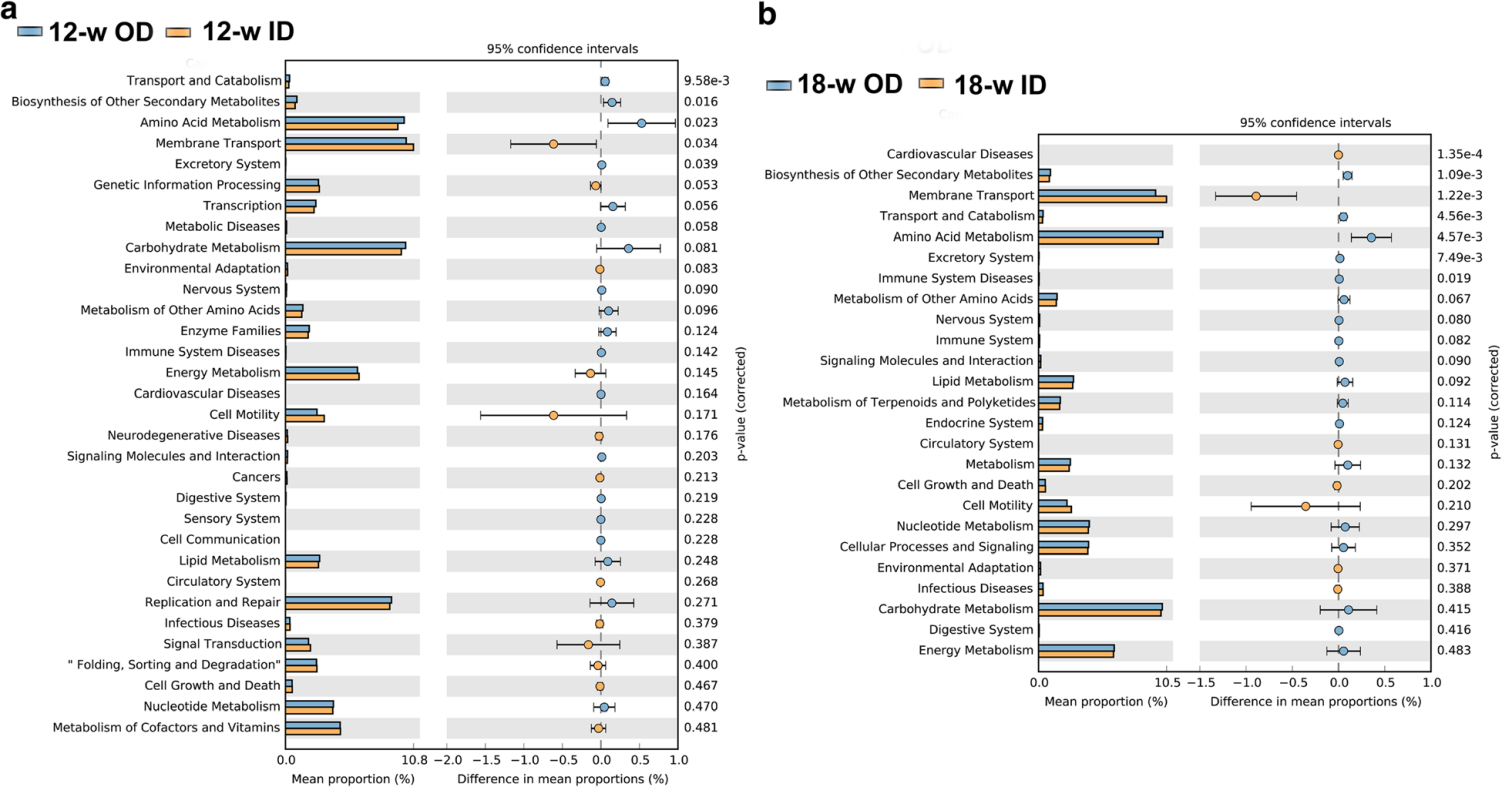
Mean proportion and their differences in predicted functional metagenomes of the cecal microbiota at KEGG level 2. Comparison of functional pathway between microbes of 12-w OD group and 12-w ID group (**a**), 18-w OD group and 18-w ID group (**b**)

Microbial function analysis through PICTUSt was conducted to determine the differences in the functions of microbiota between the OD and ID groups. Numerous functions are involved in metabolic pathways. At KEGG level 2, cecal microbiota in the OD groups have higher abundance of functions involved in amino acids metabolic pathway (Fig. 4). At KEGG level 3, cecal microbiota in the 12-w OD group have higher abundance of functions involved in metabolic pathway such as metabolism of arginine, praline, histidine, glycine, serine, threonine, alanine, aspartate and glutamate, starch and sucrose, galactose, amino sugar and nucleotide sugar, and transcription machinery, DNA replication proteins than those in the 12-w ID group. Cecal microbiota in the 18-w OD group have higher abundance of functions involved in metabolic pathway such as metabolism of glycine, serine, threonine, arginine, praline, tryptophan, phenylalanine, tyrosine, and valine, leucine and isoleucine biosynthesis, amino acid related enzymes than those in the 18-w ID group. In the OD groups, cecum contained more microbiota associated with glycosaminoglycan degradation and other glycan degradation (Additional file 4).

## Discussion

Digestion and nutrient absorption are the basic function of the intestine, where gut microbiota play an important role. These microbiota have a significant influence on intestinal tract movement, growth and development, physiological functions, and non-specific immunity [20–25]. The diversity of gut microorganism is the foundation for animals’ digestion and nutrient uptake, maintenance of biochemical functions and the intestine’s physiological functions, and promotion of the immune system’s development. Medical researches discovered that obesity is related to the changes of gut microbiota, diversity of gut microbiota apparently decreases in obese patients [26]. The results of this study show that the body weight of caged chicken was significantly higher than that of free-range groups (Table 2). This is consistent with the results of other studies [27, 28]. Figure 1 shows 34 special OTUs in OD groups and 4 special OTUs in ID groups. The diversity of cecal microbiota in the OD groups was remarkably higher than that in the ID groups (Table 1). Bailey et al. discovered that long-term stress could reduce the diversity of gut microbiota in mice [29]. Chickens raised in OD and ID groups were exposed to distinct stresses and microbiota. Chickens raised in ID groups were exposed to more stresses, such as feeding density and space [30]. Chickens raised in OD groups may be due to the earlier contact to the natural environment; thus, the diversity can be established earlier.

Host and environmental factors influence the gut microbiota. The environmental factor is more important than the host factor [31–34]. The phyla Firmicutes and Bacteroidetes dominate the intestine of mammals, followed by Fusobacteria, Proteobacteria, and Actinobacteria [35]. Bacteroidetes and Firmicutes have attracted considerable attention and are bounded to the host’s metabolism. Numerous studies investigated the probiotic effect of Bacteroidetes; they found that Bacteroidetes help the host in polysaccharide decomposition to improve nutrient utilization [36], promote immune system development, improve host’s immunity [37, 38], and maintain intestinal microecological balance [39, 40]. Results in this paper show that more (>50 %) Bacteroidetes existed in chickens in the OD groups (Fig. 2). and that the Firmicutes/Bacteroidetes ratio was smaller in the OD groups, with 0.42 and 0.62 in the 12-w groups and 0.37 and 0.49 in the 18-w groups. Research has shown that adding more dietary fiber can increase the amount of Bacteroidetes and lower the Firmicutes/Bacteroidetes ratio [41, 42]. The results show that compositions of cecal microbiota in chickens raised in two feeding modes were apparently different (Table 3, Fig. 3), especially at 12 weeks (Table 4). The difference may be attributed to the access of chickens from free-range farming to abundant microbiota in the outdoor environment; these chickens have abundance of food source and are able to intake more feedstuff containing fiber, which directly affects the composition of gut microbiota, increasing the Bacteroidetes content and lowering the Firmicutes/Bacteroidetes ratio.

Obesity is related to the distribution of gut bacteria. High ratio of Firmicutes/Bacteroidetes causes obesity because more energy has been absorbed [43]. The small intestine is mainly involved in digestion and uptake of food, while a large amount of microorganisms related to microbial fermentation exists in the large intestine, especially the cecum [44]. Food rapidly passes the front of the intestinal tract but stays for several hours in the tail end of the tract [45]. Fat deposits mainly in the large intestine [46], which is closely related to the composition of microorganisms. In chicken production, bacteria related to productivity mainly include the phylum Firmicutes, along with Bacteroidetes and Proteobacteria [47]. Researchers suggested that fat pigs have more Firmicutes but fewer Bacteroidetes, especially fewer Bacteroides that are crucial in carbohydrate degradation [48, 49]. A study revealed that free-range farming can evidently reduce the growth performance and abdominal fat of chickens [27]. However, the efficiency of converting feedstuff to energy together with the chickens’ productivity attracts increasing attention in the chickens production. In this paper, body weight of caged chickens was significantly higher than that of free-rage chickens (Table 2). We speculate that this may be due to that more Firmicutes and higher ratio of Firmicutes/Bacteroidetes in cecal microbiota improve the utilization efficiency of feed energy, of course, this needs further study.

Gut microbiota contains about 600,000 genes that are 25 times more compared with the genes in host’s genome. Therefore, gut microbiota is usually regarded as one organ of the host and creates a gut microecosystem with the host’s eucells [50, 51]. This microecosystem can execute numerous metabolic functions that alter with the change of microbiota’ composition. In this paper, numerous functions are involved in metabolic pathways, such as metabolism of amino acid, carbohydrates, energy, lipid, replication and repair, nucleotides, and cofactors and vitamins. At KEGG level 2, there are 5 significant differences (*P* < 0.05) in abundance of functional categories between OD and ID group at 12-w, whereas 7 significant differences (*P* < 0.05) were found in between at 18-w (Fig. 2). At KEGG level 3, there are 42 significant differences (*P* < 0.05) in abundance of functional categories between OD and ID group at 12-w, among them 34 in OD group was significantly higher than that in ID group (*P* < 0.05). There are 72 significant differences (*P* < 0.05) in abundance of functional categories between the OD and ID group at 18-w, among them 44 in OD group was significantly higher than that in ID group (*P* < 0.05) (Additional file 4).

Cecal microbiota of OD group at 12-w and 18-w both has higher abundance of functions involved in metabolic pathway for certain amino acids, sugar compounds. Significant difference in amino sugar and nucleotide sugar metabolism pathways were observed in 12-w groups. Utilization of amino sugar and nucleotide sugar is important in chicken metabolism and growth. Amino sugar metabolism specifically is responsible for breaking down protein present in feed to amino acids or di- or tri-peptides [52]. These were then transported from intestinal lumen to epithelial cell for energy. Nucleotide sugar metabolism on the other hand is important for purine and pyrimidine synthesis which is vital substrate for deoxyribonucleic acids derivatives. In addition, these components are also needed for producing high-energy nucleotides needed for cellular metabolism [53]. In this study, we observed that the genes responsible for amino sugar and nucleotide sugar metabolism were up-regulated in 12-w OD group compared to 12-w ID group (*P* < 0.05) (Additional file 4). This may be the outdoor chickens needs more energy due to the large amount of movement. And movement promotes muscle development, and therefore the synthesis of more body protein. In contrast, the genes related to the metabolism of amino acids, amino sugars and nucleotide sugar were up-regulated in the cecum. Previous studies show that outdoor activities could make an improvement on the meat quality. The meat of chickens with outdoor access is darker, it has more protein contents and a better water-holding capacity [54, 55]. In addition, studies have revealed that feeding chickens with probiotics can improve meat quality and increase the output of breast and leg muscles [56]. All of these are likely to be related to the changes in compositions of gut microbiota. But, more scientific research is needed to confirm this.

Based on the research above, the many metabolic functions are involved in chickens’ gut microbiota and these functions may vary because of the different compositions of gut microbiota. The compositions of chickens’ cecal microbiota varied because the chickens were raised in different feeding modes. In-depth studies on the functions of dominant gut microbiota, such as Bacteroidetes and Firmicutes and their interaction, can help us develop a special probiotics and guide us to use the special probiotics to achieve the anticipated breed goals.

## Conclusion

The composition and function of cecal microbiota in Dagu chicken under two feeding modes, free-range and cage raising are different. The cage raising mode showed a lower proportion of Bacteroidetes in cecum, but a higher Firmicutes/Bacteroidetes ratio compared with free-range mode. Cecal microbiota in free-range mode have higher abundance of functions involved in amino acids and glycan metabolic pathway. The results in this paper can provide relevant information for making strategies in raising Dagu chickens. This also provided valuable information for the study on microbiota in chicken gut.

## Additional files

Additional file 1: Diet ingredients. (DOC 38 kb)
Additional file 2: Comparisons for abundance between the 12-w OD and 12-w ID groups at the genus level. (XLS 19 kb)
Additional file 3: Comparisons for abundance between the 18-w OD and 18-w ID groups at the genus level. (XLS 19 kb)
Additional file 4: Mean proportion and their differences in predicted functional metagenomes of the cecal microbiota at KEGG level 3. Comparison of functional pathway between microbes of 12-w OD group and 12-w ID group (a), 18-w OD group and 18-w ID group (b). (TIF 3907 kb)
