# Supplementary material for: High-throughput sequencing technology to reveal the composition and function of cecal microbiota in Dagu chicken
Source: BMC Microbiol. 2016 Nov 4;16:259. doi: 10.1186/s12866-016-0877-2 (PMC5097418; doi:10.1186/s12866-016-0877-2)
Supplement: Additional file 1: — Diet ingredients. (DOC 38 kb) [file 12866_2016_877_MOESM1_ESM.doc]

**Table S1. Diet ingredients.** OD and ID groups use the same feed in two stages

| **Diet type^1^** | **7-12 W** | **13-18 W** |
| --- | --- | --- |
| Ingredient (g/kg) |  |  |
| Corn | 650 | 694 |
| Wheat bran | 60 | 140 |
| Soybean meal | 163 | 70 |
| Rapeseed meal | 40 | 20 |
| Cottonseed meal | 30 | 20 |
| Stone meal | 12 | 11 |
| Calcium phosphate | 12 | 12 |
| Salt | 3 | 3 |
| Trace elements and vitamin premix^2^ | 30 | 30 |

^1^Manufacturer: Beijing Sanyuanhefeng farming Lt. C., Beijing, China.

^2^Detailed supplementation not disclosed by the manufacturer.
