# Supplementary figures and images for: High-throughput sequencing technology to reveal the composition and function of cecal microbiota in Dagu chicken
Source: BMC Microbiol. 2016 Nov 4;16:259. doi: 10.1186/s12866-016-0877-2 (PMC5097418; doi:10.1186/s12866-016-0877-2)

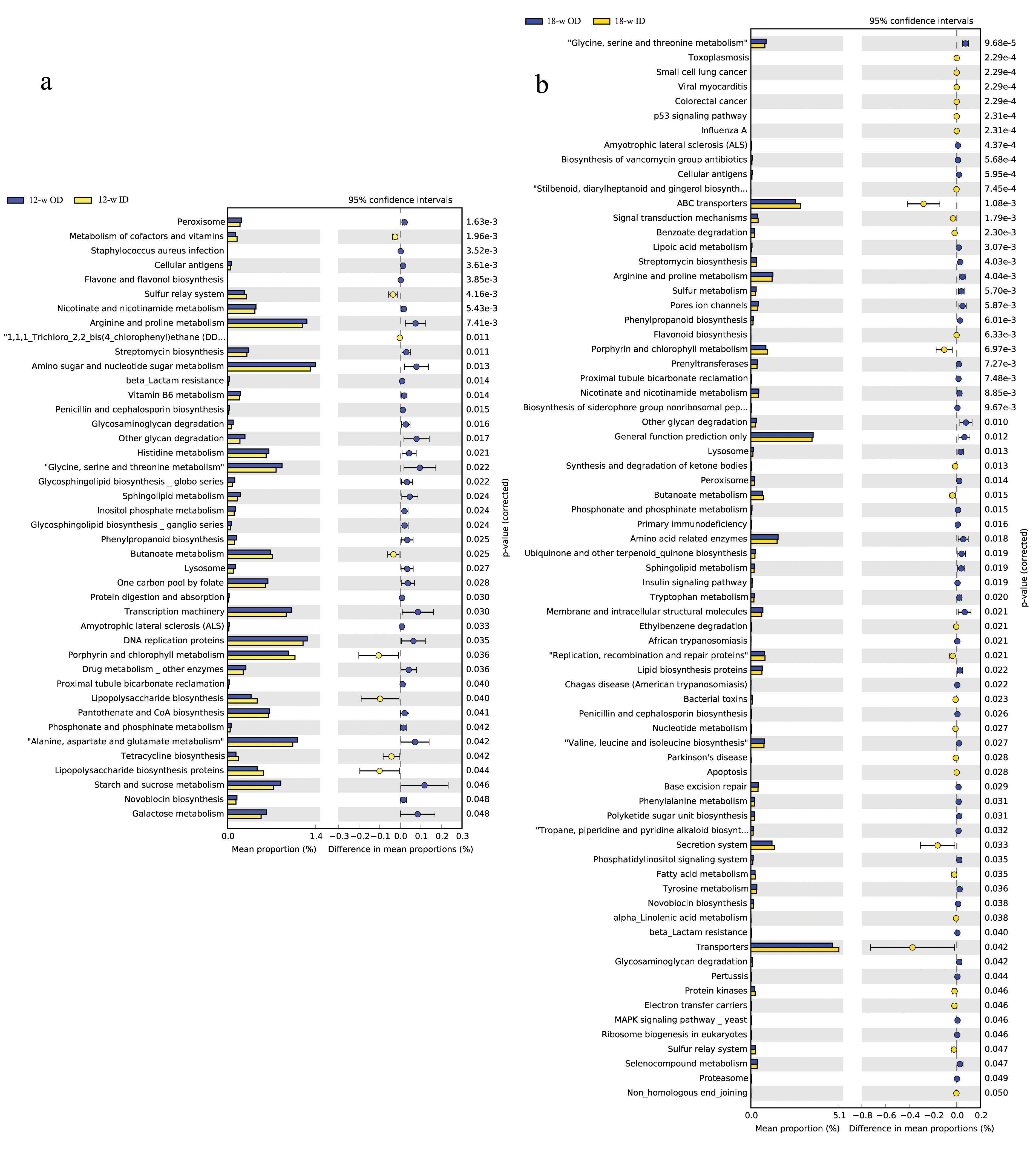

Supplement: Additional file 4: — Mean proportion and their differences in predicted functional metagenomes of the cecal microbiota at KEGG level 3. Comparison of functional pathway between microbes of 12-w OD group and 12-w ID group (a), 18-w OD group and 18-w ID group (b). (TIF 3907 kb) [file 12866_2016_877_MOESM4_ESM.tif]
